# Report of a Case Illustrating the Benefit of Laparatomy and Drainage in Desperate Cases of Purulent Peritonitis1Read before the Central New York Medical Association, at its annual meeting, held at Rochester, October 20, 1896.

**Published:** 1897-02

**Authors:** H. T. Williams

**Affiliations:** Rochester, N. Y. 52 Clinton Place


### REPORT OF A CASE ILLUSTRATING THE BENEFIT OF LAPARATOMY AND DRAINAGE IN DESPERATE CASES OF PURULENT PERITONITIS.[1]

By H. T. WILLIAMS, M. D., Rochester, N. Y.

THE case which I am about to report was referred to me July 21, 1896, by Dr. Guinan, of this city, who was called to the case only the day before. Previous to this she had been treated for several weeks by a homeopathic physician. The following history of the case was obtained :

Mrs. J. D. S., German, 38 years of age, married eighteen years ; never pregnant ; family history good ; menses regular, but scanty ; no dysmenorrhea. Had an attack of peritonitis sixteen years ago, which lasted two or three months. Always well before and since until the forepart of last June, when without apparent cause began to have pain in lower part of abdomen and pelvis, which gradually grew worse until she was confined to bed with general peritonitis.

I found the patient with a temperature of $104\frac{3}{5}°$ ; pulse, 140 to 150 ; respirations, 40 ; abdomen distended, dull on percussion over lower part of abdomen, tympanitic above ; surface of abdomen mottled from prolonged application of spirits of turpentine. Patient vomited frequently.

She was removed to Rochester City Hospital that afternoon, and as she was unable to retain food and was in a condition bordering on col-

---

1. Read before the Central New York Medical Association, at its annual meeting, held at Rochester, October 20, 1896.

lapse, I deferred operating until the next day and ordered nutrient enemata given and strychnine and brandy hypodermatically through the night. The next morning she was in a little better condition, pulse stronger, less vomiting and temperature 103.

An incision about three inches in length was made in median line of abdomen ; extensive adhesions were found between peritoneum, omentum, intestines and Fallopian tubes. Everything seemed matted together and pus was found free and in pockets throughout greater part of abdominal cavity. After breaking up some adhesions and removing some sloughs, deposits of lymph and about a pint and a half of pus, it was found that the origin of the trouble was in the Fallopian tubes, and these were so adherent and disorganised that removal of them was out of the question. The abdomen was flushed with several gallons of salt solution, an opening was made through vagina posterior to uterus and a large-sized rubber tube passed from abdominal opening into vagina and another one from opening in abdomen deep into left side of pelvis. Iodoform gauze was packed around tubes, usual dressing applied and patient put to bed.

In a few hours her temperature had dropped to $101\frac{2}{5}°$ ; pulse, 140, respiration, 36. Next day temperature was $100\frac{4}{5}°$; pulse, 120. The temperature never rose above 100° after this. *Considerable* pus discharged from tubes for several days and then became rapidly less each day. Bowels were moved every day by either laxatives or enemata. The seventh day after operation the nurse noticed fecal matter coming through the vagina, and the next day the bowels moved entirely through the vagina. The drainage-tubes were then withdrawn and with forceps several large sloughs were easily removed. These sloughs extended in different directions deep into the pelvis, after removal of which urine and fecal matter came through abdominal opening and pus through urethra. The abdominal cavity was packed with iodoform gauze, which had to be changed several times a day and all parts irrigated with 2 per cent. creoline solution. A catheter was kept in bladder and the creoline solution was thrown through this and washed through abdominal opening. Fecal matter and urine were discharged entirely through vagina and abdominal opening. After this state of affairs had existed for about a week, it occurred to me that if the anus could be kept patulous the bowels might possibly move through it. Acting on this idea I inserted a large rubber tube through anus and about eight inches into bowel, and the vagina and abdominal opening kept firmly packed with iodoform gauze. This procedure was extremely satisfactory. From this time on, the bowels moved entirely through the tube and in ten days the fecal fistula had entirely healed. The tube was removed and since that time her bowels have moved naturally. One thing noticeable was the pain caused by the presence of the tube in the rectum. The patient begged for its removal several days before it was thought safe to remove it, and seemed to suffer more from this tube than from anything else during her illness.

After a week or so, less pus discharged through catheter in bladder and it was noticed that considerable urine came through it, and less urine through the abdominal opening.

The catheter was left in bladder, being frequently removed and cleansed, and bladder irrigated with creoline or boracic acid solutions for six weeks, when discharge of urine from wound having nearly ceased the catheter was removed. Since then she has urinated naturally every four to six hours. Abdominal opening is now nearly closed, but occasionally, once in two or three days, when bladder is quite full, a few drops of urine escape through abdominal fistula. No urine has escaped in that way for the past five or six days. Patient is up and around, eats and sleeps well and is getting fat, in fact is nearly well. Has not menstruated since operation.

The case is interesting in demonstrating what may be accomplished by free drainage and irrigation in desperate cases of purulent peritonitis, even where serious complications exist.

52 Clinton Place.

